# 

**Published:** 1860-09

**Authors:** 


**VOLUME XIV.**

### DENTAL REGISTER,

### OF THE WEST.

This volume will be issued in 6 months, commencing with the July Number, and closing with the December Number.

The price is **ONE DOLLAR,** for this volume.

**NOW IS THE TIME TO SUBSCRIBE.**

*Send on your Dollar and have your name enrolled.*

**TERMS - - STRICTLY CASH, IN ADVANCE.**

---

A FEW ADVERTISEMENTS WILL BE INSERTED AS FOLLOWS.

| | |
|---|---|
| One Page, - - - - - | $30. |
| Half Page, - - - - | $20. |
| Quarter Page, - - - - | $15. |

